# The influence of acetyl phosphate on DspA signalling in the Cyanobacterium *Synechocystis *sp. PCC6803

**DOI:** 10.1186/1471-2180-5-47

**Published:** 2005-08-02

**Authors:** S Shawn Morrison, Conrad W Mullineaux, Mark K Ashby

**Affiliations:** 1 Department of Basic Medical Sciences, Biochemistry Section, the University of the West Indies, Mona Campus, Kingston 7, Jamaica; 2 Department of Biology, University College London, Darwin Building, Gower Street, London WC1E 6BT, UK; 3 School of Biological and Chemical Sciences, Queen Mary, University of London, Mile End Road, London E1 4NS, UK

## Abstract

**Background:**

The *dspA *(*hik33*) gene, coding for a putative sensory histidine kinase, is conserved in plastids (*ycf26*) and cyanobacteria. It has been linked with a number of different stress responses in cyanobacteria.

**Results:**

We constructed an insertional mutant of *dspA *(*ycf26) *in *Synechocystis *6803. We found little phenotypic effect during nitrogen starvation. However, when the mutation was combined with deletion of the *pta *gene coding for phosphotransacetylase, a more significant phenotype was observed. Under nitrogen starvation, the *pta/dspA *double mutant degrades its phycobilisomes less than the wild type and still has about half of its chlorophyll-protein complexes.

**Conclusion:**

Our data indicates that acetyl-phosphate-dependent phosphorylation of response regulator(s) overlaps with DspA-dependent signalling of the degradation of chlorophyll-protein complexes (and to a lesser extent phycobilisomes) in *Synechocystis *6803.

## Background

The assembly, composition and maintenance of photosynthetic pigment-protein complexes are regulated by many different factors including light quality, light intensity, temperature, water availability and nutrient status [1]. Microorganisms have diverse mechanisms for sensing and acclimating to environmental parameters. In bacteria, two-component signal transduction pathways form a major part of the signalling machinery, mediating adaptive responses to a broad range of environmental stimuli [2-4]. In response to such stimuli, a sensor kinase autophosphorylates an internal histidine residue, and then transfers the phosphate to a conserved aspartate residue on the receiver domain of its cognate response regulator. The phosphorylation of the response regulator often leads to changes in gene transcription [5].

Response regulators are generally phosphorylated by their cognate sensor kinases, but phosphorylation can also be mediated through low molecular mass phosphate donors, such as acetyl phosphate, phosphoramidate (NH_2_PO_3_) and carbamyl phosphate [6-9]. The *pta *gene encodes phosphotransacetylase which catalyses the production of acetyl phosphate, the activated acetate intermediate that occurs during conversion of acetyl-CoA to acetate [10]. Chamnongpol & Groisman [11] identified acetyl phosphate as the phosphate donor for a mutant version of the response regulator PhoP (PhoP*) and showed that inactivation of the *pta *gene abolished PhoP*-mediated transcription. Most response regulators can be phosphorylated by acetyl phosphate *in vitro *[8], but at physiological concentration this may not be significant depending on the *K*_*m *_for each individual response regulator for acetyl phosphate. It may be that acetyl phosphate maintains a low level of phosphorylation for most response regulators. The *pta *gene has been identified in *Synechocystis *6803 (slr2132) and *Crocosphaera watsonii *by homology to other bacterial *pta *sequences (up to 47% sequence identity by BLASTP), however *pta *has not been identified in the genome sequence of the other cyanobacteria.

The gene *dsp*A (*ycf*26) codes for a putative sensory histidine kinase that is conserved in plastids and cyanobacteria. The deduced amino acid sequence of DspA contains 663 amino acids in *Synechocystis *sp. PCC 6803 (*Synechocystis *6803). DspA has a HAMP [12] and a PAS sensor domain [13]. We have also identified a possible additional sensory domain in Ycf26, which will be discussed below. All cyanobacterial genomes sequenced to date contain an ORF encoding a Ycf26 orthologue and *ycf26 *is also the only histidine kinase gene present in the plastid genomes of the red algae *Porphyra purpurea, Gracilaria tenuistipitata *and *Cyanidium caldarium *[14]. This suggests that it performs a key role in the regulation of the level of photosynthetic pigment-protein complexes.

The probable orthologue for Ycf26 in the cyanobacterium *Synechocystis *6803 is an ORF designated sll0698 in the CyanoBase genome database  which has also been called Hik33 [15] and DspA [16]. It was identified as a factor that confers resistance to a variety of herbicides and was suggested to be a histidine kinase involved in chemical sensing [16]. It was found to be involved in gene expression (including photosynthesis-related genes), demonstrating characteristics of a sensor that can detect a decrease in membrane fluidity, and is involved in the perception and transduction of salt stress and low temperature signals in *Synechocystis *6803 [15,17,18]. Deletion of this open reading frame leads to global changes in gene expression [19,20] and in addition a potential link has been identified between Hik33 and Rre31 (RpaA). In another cyanobacterium, *Synechococcus *sp. PCC7942 (*Synechococcus *7942), the probable orthologue for Ycf26 was shown to regulate phycobilisome degradation during nutrient starvation and in high light. The gene was therefore designated *nbl*S (non-bleaching mutant sensor) [21]. DspA is also important for the survival of *Synechocystis *6803 when grown in high-light [22]. One possible cognate response regulator (NblR) has been identified in *Synechococcus *7942 for NblS, however, no orthologue for NblR has been identified in *Synechocystis *6803 (MK Ashby & J Houmard, unpublished) and it is likely that NblS also interacts with other response regulators [21]. NblS has been proposed to integrate redox and light signals and may influence other signalling pathways involved in acclimation responses [21]. Thus it appears that the orthologues of DspA in other cyanobacteria have different but overlapping functions.

A role for the sll0698 gene product in controlling the degradation of the photosynthetic apparatus during nutrient stress has not yet been demonstrated in *Synechocystis *6803. We have constructed an insertional deletion mutant in this ORF, which we will refer to as *dspA*. We show that the inactivation of *dspA *has little phenotypic effect under nitrogen starvation, unless the mutation is combined with deletion of the *pta *gene. In the double mutant there is a dramatic effect on the degradation of chlorophyll-protein complexes under nitrogen starvation, which is not seen in either of the single mutants, however phycobilisomes are degraded, though not to the same level as in wild type. We conclude that acetyl phosphate masks DspA signalling under our laboratory conditions. The results indicate that the degradation of photosynthetic complexes during nutrient stress is controlled by multiple inputs, including acetyl phosphate level.

## Results

### Analysis of cyanobacterial and plastid deduced Ycf26 protein sequences

**Figure 1:**
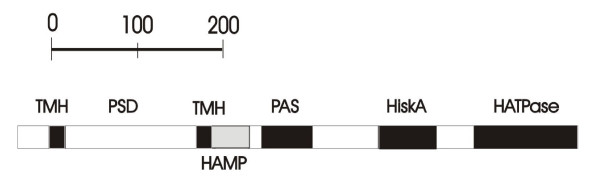
**Cartoon of the domain structure of Ycf26 (NblS, DspA)**. HAMP – histidine kinase and methyl-accepting proteins; HisKA and HATpase – catalytic domains of histidine kinase; PAS – period protein, aryl hydrocarbon receptor nuclear translocator protein and single-minded protein; PSD – Putative sensor domain; TMH – Trans membrane helix;

The deduced amino acid sequences for Ycf26 from 15 available cyanobacterial annotated genome sequences and the red algal species *Porphyra purpurea, Gracilaria tenuistipitata *and *Cyanidium caldarium *were analysed by Pfam and SMART. This identified a HAMP, PAS (low confidence in *Cyanidium*), HisKA and HATPase_c domain (Figure 1). SMART and Interpro also identified two potential transmembrane helical regions upstream of the HAMP domain (33–55 and 201–223 in 6803). The identification of transmembrane sequences by SMART is with an accuracy of 97–98% [23]. Because the 145 amino acid region that is flanked by the two transmembrane regions is highly conserved in all 15 cyanobacterial sequences and *Porphyra *(see additional figure online and Prodom Family PD339187 [24]) and it is adjacent and upstream of a HAMP domain (which is known to in most cases be actively involved in transmission of signals from a periplasmic signalling domain to the cytoplasmic portion of the protein [12,25,26]), we think it probably functions as a periplasmic sensor. We have not been able to detect homologous sequences in any other protein sequence, suggesting that it is unique to Ycf26 (DspA/NblS). The PAS sensor domain may bind a redox sensitive cofactor [13] that could potentially report on the metabolic state of the cell. Phylogenetic analysis (Ashby, MK and Houmard, J unpublished) and ClustalW alignments of these sequences and the alignment scores indicate that all these proteins (except *Cyanidium caldarium *and *Gracilaria tenuistipitata*) are probably orthologues (see supplementary data online). This is supported by Tu *et al*., [19] who were able to rescue their *dspA *null mutant in *Synechocystis *6803 with the *nblS *gene from *Synechococcus *7942.

### Phenotype of the *pta*, *dspA *and *pta/dspA *mutants under nutrient-replete conditions

Mutants were routinely characterised by whole cell absorption spectra, which can be used to give an estimate of the amount of chlorophyll and phycocyanin per cell [27]. Mutants were further characterised by 77 K fluorescence emission spectra. These spectra give an indication of changes in the PSII:PSI ratio [28].

**Figure 2:**
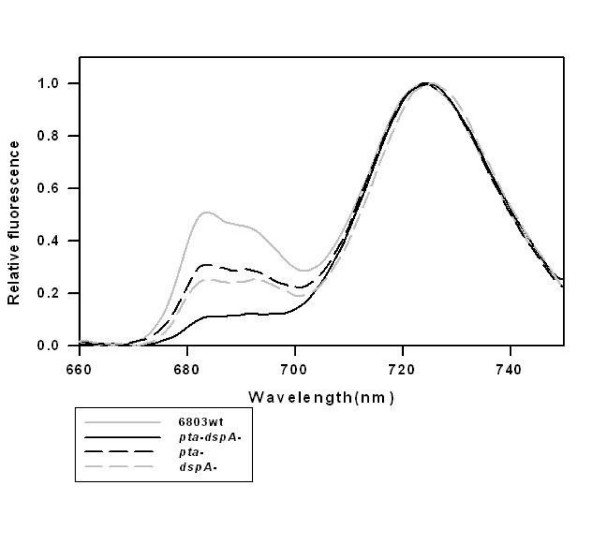
**77 K fluorescence emission spectra for cells grown under nutrient-replete conditions**. *Synechocystis *6803 wild-type and mutants. Excitation is at 435 nm (chlorophyll excitation) and spectra are normalised to the PSI fluorescence emission peak at 725 nm. Cells grown under moderately high light (80 μmol m^-2 ^s^-1^).

When cells were grown under low light (white light at 10 μmol m^-2 ^s^-1^) the absorption spectra were similar, indicating little difference in the chlorophyll and phycocyanin content of the cells (data not shown). When cells were grown under higher light (80 μmol m^-2 ^s^-1^), only slight differences in absorption spectra and Chl/PC ratios were seen (Table 2). However low-temperature fluorescence spectra recorded with chlorophyll excitation revealed changes in the ratio of PSII/PSI (Figure 2). These spectra show peaks at 685 nm and 695 nm (both from PSII) and 725 nm (PSI) [28,29]. Spectra have been normalised to the PSI fluorescence emission, as their absolute amplitudes are unreliable [28]. When cells were grown at 80 μmol m^-2 ^s^-1^, the *dspA *and *pta *single mutants show a lower PSII/PSI ratio than the wild-type, and this difference was more pronounced in the *pta/dspA *double mutant (Figure 2). A similar pattern was seen in cells grown at 10 μmol m^-2 ^s^-1^, but the differences were less pronounced (not shown).

### Phenotype of the *pta*, *dspA *and *pta/dspA *mutants after nitrogen starvation

An *nblS *mutant of *Synechococcus *7942 shows a pronounced phenotype under nitrogen starvation. In the wild-type, cells gradually lose their photosynthetic pigments under these conditions. However, an *nblS *mutant is bleached to a much lesser extent, retaining its phycobilisomes in particular [21]. We therefore looked at the pigmentation of our *Synechocystis *6803 mutants during growth in modified BG11 medium supplemented with glucose (to help maintain the viability of the cells), but containing no NaNO_3_.

**Figure 3:**
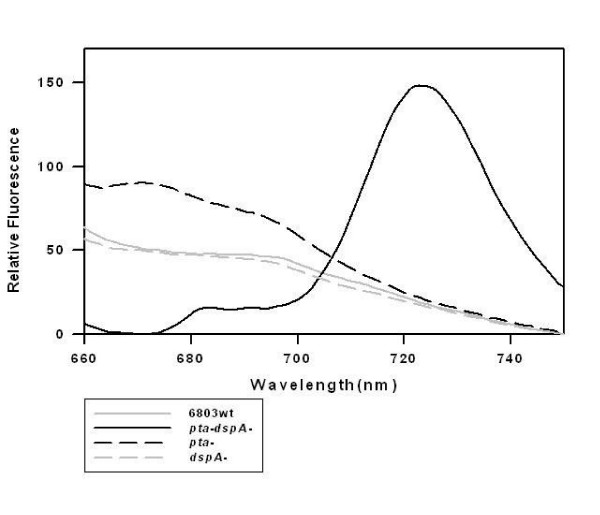
**77 K fluorescence emission spectra for cells after growth in nitrate-depleted medium**. *Synechocystis *6803 wild-type and mutants grown for 3 days in these conditions. Excitation is at 435 nm.

After three days nitrogen starvation in moderate light (80 μmol m^-2 ^s^-1^), the wild-type and the *dspA *and *pta *single mutants were almost completely bleached (Table 2). We could detect no significant differences (less than 5%) in the rate of bleaching during nitrogen starvation in the three strains (data not shown). By contrast, the *pta/dspA *double mutant retained significant levels of pigment (Table 2). Both the chlorophyll and phycocyanin per cell dropped somewhat compared to the start of the treatment, but loss of phycocyanin was much greater (Table 2). Thus only the *pta/dspA *double mutant shows a 'non-bleaching like' phenotype: Chlorophyll concentration decreases by a factor of about two and that of phycocyanin by about five. Figure 3 shows low-temperature fluorescence emission spectra for the *pta/dspA *double mutant grown after 3 days nitrogen starvation. Whether with or without nitrate, it exhibits a similar spectrum meaning that the starved cells retain both PSI and PSII. The starved *pta*^-^*/dspA*^- ^cells also retained significant levels of oxygen evolution (data not shown). Thus the *pta/dspA *double mutant retains its photosynthetic apparatus during nitrogen starvation, in contrast to the wild-type and both single mutants, where nearly all pigment is lost.

## Discussion

### A potential new sensor domain in DspA/Ycf26

Ycf26 may have a periplasmic sensor domain, which could play an important role in sensing stress either directly or indirectly (Figure 1). The putative periplasmic sensor and PAS domain may combine to enable Ycf26 to sense a wide variety of stress conditions [[15,17-19,21,22] & this work]. It remains to be determined whether DspA senses any of these signals directly or via other proteins or phosphorelays and the precise way in which these sensor domains interact. This apparent central role of Ycf26 in the regulation of gene expression in response to a number of different stresses is supported by its universal occurrence in the 15 available cyanobacterial genome sequences, particularly the 3 *Prochlorococcus *species which only have 4–6 histidine kinases in their genome ([30], MK Ashby & J Houmard, unpublished).

### Roles of DspA and acetyl phosphate in cell signalling

**Figure 4:**
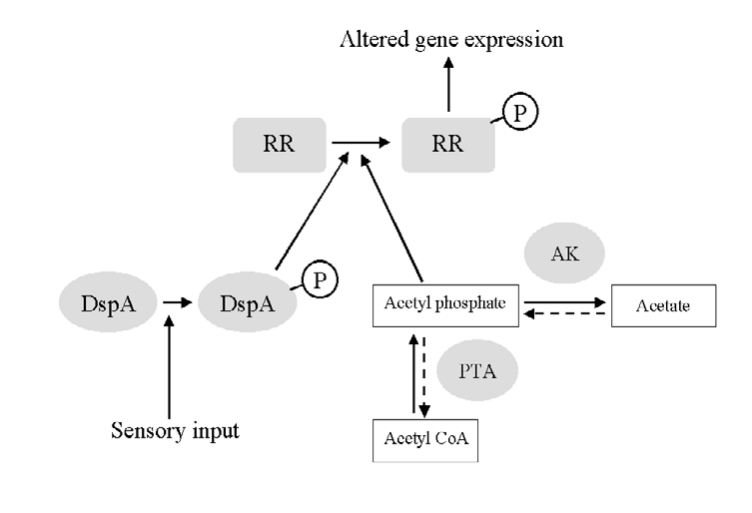
**Simplest model that fits the data for information flow during DspA signalling**. Acetyl phosphate and DspA can both act as phosphodonors to one or more response regulators. Under nitrogen starvation, phosphorylation by either acetyl phosphate or DspA alone is sufficient to saturate the response. Thus *pta *or *dspA *single mutants behave like the wild-type, and only the *pta*^-^*dspA*^- ^double mutant has a distinctive phenotype. Under other conditions the steady-state phosphorylation level of the response regulators (or hybrid kinases) is not saturated either by acetyl phosphate or by DspA alone. Then *pta *and *dspA *deletions have additive effects. Both single mutants show a phenotypic change, but a stronger effect is seen in the double mutant. RR: response regulator; AK: acetate kinase; PTA: phosphotransacetylase.

We have constructed *Synechocystis *6803 mutants deficient in the *pta *gene coding for phosphotransacetylase and the *dspA *gene coding for the DspA sensory histidine kinase (otherwise known as Ycf26 or NblS), and a double mutant deficient in both genes. We have used the mutants to examine the possible roles of DspA and acetyl phosphate in controlling cell responses to nitrogen starvation. We find strong evidence that DspA and acetyl phosphate act cooperatively as signalling inputs into pathways controlling photosystem stoichiometry under nutrient-replete conditions, and photosystem degradation during nitrogen starvation. Under nutrient-replete conditions, the *dspA *and *pta *single mutants both show a decreased PSII/PSI ratio (Figure 2). The PSII/PSI ratio becomes even lower in the *pta/dspA *double mutant, indicating additive effects of the two mutations. Since acetyl phosphate is known to act as a phosphodonor to response regulators [11], we propose that PSII/PSI ratio is influenced by gene expression controlled by one or more response regulators (or hybrid kinases) whose phosphorylation level is controlled by phosphotransfer both from DspA and from acetyl phosphate. This scheme (in its simplest form) is illustrated in Figure 4.

The effects seen under nitrogen starvation are even more striking. After 3 days' nitrogen starvation under moderate light, wild-type cells are virtually devoid of pigment, indicating almost complete loss of the phycobilisomes and the chlorophyll-protein complexes (Table 2). In this case, the *dspA *and *pta *single mutants both behaved in the same way as the wild-type, but the *pta/dspA *double mutant retained a significant proportion of its photosynthetic complexes, in particular the chlorophyll-protein complexes. Therefore the *pta/dspA *double mutant is clearly deficient in a signalling pathway required to initiate degradation of these complexes under these conditions. Again, we suggest that this pathway involves gene expression controlled by one or more response regulators whose phosphorylation level is determined by phosphotransfer from both DspA and acetyl phosphate (Figure 4). In this case the double mutation is needed to produce a detectable phenotypic effect, suggesting that either DspA or acetyl phosphate alone are sufficient to maintain high enough levels of phosphorylation of the relevant response regulator(s) (Figure 4). The fact that the phenotypic difference is seen only in the double mutant very strongly suggests that DspA and acetyl phosphate are acting as phosphodonors to the same response regulator(s). Again, it appears that a high level of response regulator phosphorylation promotes the degradation of the photosynthetic complexes.

### Acetyl phosphate as a potential global regulator of gene expression in *Synechocystis *6803

In *E. coli *the concentration of acetyl phosphate has been shown to be dependent on the metabolic state of the cell as well as the growth phase, carbon source, pH and temperature [8,31,32], therefore acetyl phosphate could potentially serve as a global sensor of the physiological state of the cell in *E. coli*. Acetyl phosphate is known to phosphorylate several RR's, including CheY, NR_I_, PhoB and OmpR [8]*in vivo*, but its ability to phosphorylate response regulators *in vivo *depends on the *K*_*m *_of the response regulator for acetyl phosphate, which in many cases is too high to allow phosphorylation [33]. In the four systems studied in one investigation of crosstalk between two component systems, one, the Uhp system was shown to be activated by acetyl phosphate when *E. coli *is grown on pyruvate [34]. There have been no comparable studies in cyanobacteria, but it seems likely that the level of acetyl phosphate would change in response to physiological stress, in particular nutrient deprivation. If this is the case, it seems likely that the *K*_*m *_of at least some response regulators could be tuned to the physiological concentrations of acetyl phosphate in *Synechocystis *6803, allowing the level of acetyl phosphate to be part of the signalling process. The level of pigment complexes in the *pta *mutant was close to normal in nutrient replete conditions (table 2), indicating that the level of acetyl phosphate is not having an effect on overall expression of photosynthetic gene expression via a number of response regulators, but we cannot rule out that the cumulative effect of *pta*^- ^and *dspA*^- ^that we observe in nutrient depleted conditions may be influenced by a reduced phosphorylation of response regulator(s) other than those phosphorylated by DspA.

### Role of DspA in initiating degradation of photosynthetic complexes during nutrient stress in *Synechocystis *6803

NblS (DspA homologue) was originally identified in *Synechococcus *7942 as a factor important for initiating the degradation of the photosynthetic complexes (in particular the phycobilisomes) during nitrogen starvation [21]. In *Synechocystis *6803 there is strong evidence for DspA as a global regulator controlling the expression of many genes involved in photosynthesis, carbon metabolism and stress responses [15,17-19,21,22]. However, *dspA *mutations in *Synechocystis *6803 have not previously been shown to induce a non-bleaching phenotype like that seen in *Synechococcus *7942. Here we have shown that such a phenotype can be seen in the *dspA *mutant of *Synechocystis *6803, provided that the *pta *gene is also deleted. The phenotype is still not identical to that seen in *Synechococcus *7942, however, since in *Synechocystis *6803 it is not only the degradation of the phycobilisomes which is affected but also the degradation of chlorophyll-protein complexes (Table 2).

## Conclusion

It is clear that, under our conditions, DspA is a sensor for nitrogen starvation in association with the level of acetyl phosphate, leading to photosystem degradation, since this response occurs similarly in the wild-type and in the *dspA *single mutant (Table 2). However, it is unlikely to be coincidental that non-bleaching phenotypes are associated with *nblS*/*dspA *mutations both in *Synechococcus *7942 and in *Synechocystis *6803. We suggest that there must be other, as yet undetermined, conditions in which acetyl phosphate levels in wild-type cells are low. Under such conditions, DspA activity will become a physiologically important factor controlling the degradation of the photosynthetic complexes. The presence of Pta in *Synechocystis *6803 may reflect its more flexible metabolic lifestyle compared to other cyanobacteria.

## Methods

### Strain and culture conditions

All strains used are derivatives of the glucose-tolerant strain of *Synechocystis *6803 [35] and are listed in Table 1. All strains were grown in BG-11 medium [36] supplemented with 10 mM sodium bicarbonate and 12 mM sodium thiosulphate at 30°C in an illuminated shaking incubator (New Brunswick) under white light. For nutrient deprivation studies, BG-11 medium lacking NaNO_3 _but supplemented with 5 mM glucose was used, this also served as the wash medium. Illumination was at 80 μEm^-2^s^-1 ^unless otherwise indicated. Cultures for nitrogen starvation experiments were grown in BG-11 with glucose at a low cell density for at least 3 days prior to nitrogen starvation. Erythromycin and kanamycin were added to media or plates when required at a final concentration 50 μg ml^-1^.

### Sequence analysis

Deduced amino acid sequences were obtained form Cyanobase [37], the National Centre for Biotechnology Information (NCBI) [38] and the DOE Joint Genome Institute [39]. Protein to protein BLASTP 2.2.6/3 searches were performed at NCBI and Cyanobase websites. Protein sequences were analysed by Pfam [40,41], SMART [23,42] and Interpro [43]. CLUSTALW alignments [44] were performed at the European Bioinformatics Institute [45].

### Molecular biology

Routine DNA manipulations were performed as in [46] using reagents purchased from QIAGEN, New England Biolabs, Inc., Promega and Sigma. Genomic DNA from *Synechocystis *6803 was prepared as described by Porter [47]. The primers used for PCR amplification of the *dspA *gene had the sequences 5'-gcgagctcttctgtgtccaatccaacg and 5'-gcggtaccatggattgatacacggccag. The same primers were used to monitor segregation in the transformant. The primers used for PCR amplification of the *pta *gene had the sequences 5'-GCGAGCTCCCTTTATTTAAGCACCACC and 5'-GCGGTACCTTGCAAAGCTGTAATTACCACC and were also used to monitor segregation in the transformants. Primers were purchased from Invitrogen Technologies.

### Construction of *dspA *and *pta *mutants of *Synechocystis *6803

The *pta *open reading frame (slr2132 in the Cyanobase database) and the *dspA *open reading frame (sll0698) were amplified by PCR from *Synechocystis *6803 DNA, using primers shown above. The PCR products were cloned into pBluescriptII KS +. The slr2132 sequence was cut at a *Pst*I site at position 872 in the coding sequence, and an erythromycin resistance cassette (pBSEmEco1) was ligated in. The sll0698 sequence was cut at an *Eco*RI site at position 1384 in the coding sequence, and a kanamycin resistance cassette (pUC4K) was ligated in [48,49]. Slr2132 is 265 bp downstream of slr1888 and it is probably transcribed on an mRNA encoding only Pta, so its interruption should not have any polar effects. Sll0698 is upstream of two putative transposases sll0699 and sll0700. The glucose-tolerant strain of *Synechocystis *6803 was transformed with the slr2132::Em^R ^construct as described by Porter [47]. After several rounds of growth on erythromycin plates, PCR was used to check the insertion. This confirmed that the wild-type slr2132 locus had been completely replaced by the slr2132::Em^R ^locus, and hence that the *pta *gene had been completely inactivated generating a fully-segregated *pta *mutant. A similar procedure was followed with the sll0698::Km^R ^construct, which was used to transform both the wild-type and the *pta *mutant, to generate a sll0698 single insertional mutant (*dspA*) and a sll0698:slr2132 double insertional mutant (*pta/dspA*). These mutants were shown to have segregated after six rounds of streaking to single colonies on kanamycin (plus erythromycin for *pta *double mutants) plates as has been observed by Hsiao *et al. *[22], though without glucose in this work.

### Procedure for initiating nutrient deprivation

50 ml cultures were grown to mid-log phase in BG11 medium. Each culture was then harvested by centrifugation, resuspended in 30 ml of wash medium, harvested a second time and resuspended in 10 ml of BG11 medium supplemented with glucose but lacking NaNO_3_. This was then divided into two 5 ml portions, which were then used to inoculate parallel 20 ml cultures.

### Pigment content analysis

Fluorescence emission spectra were measured at 77 K in a Perkin-Elmer LS50 luminescence spectrometer equipped with a liquid-nitrogen sample holder. Cells were harvested by centrifugation and resuspended in growth medium to a chlorophyll *a *concentration of 5 μM [28]. Re-absorption of emitted fluorescence was negligible at the cell concentrations used. The samples were not mixed with glycerol, as this alters the low temperature fluorescence emission spectrum [29]. The samples were then injected into silica tubes (2 mm internal diameter) and dark adapted for 5 minutes. Samples were rapidly frozen by immersion in liquid nitrogen. Excitation and emission slit widths were 5 nm. Chlorophyll *a *was excited at 435 nm: light at this wavelength is not significantly absorbed by the phycobilisomes [50]. Chlorophyll *a *concentration was estimated from the absorption of methanol extracts at 665 nm [51]. Spectra from different samples were normalised to the PSI peak (emission at 725 nm). Cell absorption spectra were recorded with an Aminco DW2000 spectrophotometer. The concentrations of chlorophyll and phycocyanin were estimated from the spectra using the equations of Myers *et al*. [27]. Cell numbers were estimated from the optical density at 750 nm, calibrated using a hemocytometer.

## Abbreviations

aa – amino acids; Dsp – drug sensory protein, HAMP – histidine kinases, adenyl cyclases, methyl binding proteins and phosphatases; NblS – non-bleaching mutant sensor, PAS – PER-ARNT-SIM; RR – Response regulator.

## Authors' contributions

SSM performed the spectroscopy, analysed the segregation of the mutants and drafted the manuscript. CWM supervised SSM at UCL and drafted the manuscript. MKA conceived the study, constructed the interposon mutants and drafted the manuscript.

## Supplementary Material

Additional File 1 ClustalW alignment of NblS (DspA, Ycf26) putative amino acid sequences. Click here for file

## Figures and Tables

**Table 1:** Bacterial strains and plasmids used

**Strain**	**Description**	**Reference/Source**
*Synechocystis *6803	Glucose-tolerant 'wild-type' strain	[35]
*pta*	ORF slr2132 inactivated by insertion of Em^R ^gene into *Pst*I site at position 1,251,313 of the genome (872 bp into the coding seq of slr2132).	This study
*dspA*	ORF sll0698 inactivated by insertion of Km^R ^gene into *Eco*R1site at position 125,060 of the genome (1384 bp into the coding seq of sll0698).	This study
*ptadspA*	ORF sll0698 inactivated in *pta*	This study
pBluescriptII KS +	Multipurpose cloning vector with Amp^R^.	Stratagene
pBSEmEco1	Cloned Em^R ^gene cassette.	[48,49]
pUC4K	Cloned Km^R ^gene cassette	Stratagene

**Table 2:** Pigment contents of *Synechocystis *6803 wild-type and mutants. Data before and after nitrogen starvation for 3 days at 80 μmol m^-2^s^-1 ^as described in Experimental Procedures. Pigment concentrations and cell densities were estimated from absorption spectra. Values given are the means of three replicates, with standard deviations. Asterisks indicate pigment concentrations that were too low to be accurately determined. All media contained glucose.

**Strain**	**Chlorophyll/Cell **(molecules × 10^6^)		**Phycocyanin/Cell **(molecules × 10^6^)		**Chlorophyll/phycocyanin**	
	**+nitrate**	**-nitrate**	**+nitrate**	**-nitrate**	**+nitrate**	**-nitrate**
Wild-type	13.6 ± 2.1	*	5.6 ± 1.4	*	2.4 ± 0.1	*
*pta*	12.6 ± 0.4	*	4.6 ± 1.5	*	2.7 ± 0.1	*
*dspA*	13.8 ± 0.1	*	5.9 ± 0.5	*	2.4 ± 0.1	*
*pta/dspA*	15.8 ± 0.9	7.8 ± 0.5	5.9 ± 0.5	1.0 ± 0.2	2.7 ± 0.2	7.8 ± 2.3
