# Supplementary material for: The influence of acetyl phosphate on DspA signalling in the Cyanobacterium Synechocystis sp. PCC6803
Source: BMC Microbiol. 2005 Aug 2;5:47. doi: 10.1186/1471-2180-5-47 (PMC1192802; doi:10.1186/1471-2180-5-47)
Supplement: Additional File 1 — ClustalW alignment of NblS (DspA, Ycf26) putative amino acid sequences. [file 1471-2180-5-47-S1.doc]

6803sll0698 1 -------------MGTSVSNPTAILQTMQGFLRKWWSEFNLQTRLMAAATLVVSLLMSGL
cwat197946 1 --------------------LFNLLSQIKEILRRWWSEFTLQTKLMAAATLAVSLFMSGL
7120alr3511 1 --------------------MLALLKTIREAIANWWSEFTLQTKLLAAATLVVSLVMSGL
AvarZP00161981 1 --------------------MLALLKTIREAIANWWSEFTLQTKLLAAATLVVSLVMSGL
NPUNNpR3716 1 --------------------MLTLLKTIRHAIASWWSEFTLQTKLLAVATLVVSLVMSGL
TERY00073906 1 --------------------MLPLLKKIGEIIANWWSEFTLQSKLMAVITLIVSLLMSSL
7942126282 1 --------------------MLALWQQLRDTLTRWWADFTLQTKLMAIATLVVSLIMSGL
TBP1tlr0437 1 ------------MVVLKTETTQGSWSDKLRGIARWWSEFKIQTRLMATATLVVSIIMSGL
SS12Pro1422 1 -MTTNNINQIEPTQEEERSDDKTSHPGLKPLINKWWDEFSLRAKLLAIATLIVSLLMSGI
MED4PMM1341 1 MIESNEPNKIKLNPSKKFIDENNPD-HWSSRVLTWWSGFSLRTKLLAIATLVVSLLMTGI
960533/318 1 -MSSGSGAAIADWALPPNGKPPGEDQNLWDRITAWWAEFTLQTKLLAIATLVVSLMMTGI
990231/129 1 -MSSESGAVIADWALPPNGGPPTDDNTLWKRIAAWWAEFTLQTKLLAVATLVVSLMMTGI
SYNW0551 1 -MTT---PATADWALPQPGGPDPGSDGIWRRIVAWWAEFSLQTKLLAVATLVVSLVMTGI
9313PMT1417 1 -MASSTSQAIAEWAPPGDGQPPPEPPGWGERIYLWWAEFSLQTKLLAIATLVVSLLMTGV
7421gll0055 1 -------MGIWQNDVIAVDITNASALNFSKNLQRWFSEFRIQTRLLAAAALIISLVLASF
Ppurpurea 1 -----MFSFRNQQVLTFVSSLSTFVTIILNHLKKWWSDVTLRTRLMAMTTLMVSLLMSSL
Gtenuistipitata 1 -----------------------MYSKILEISSKFDFKINFS-RFIVFITLMISVIMSSL
consensus 1 m i wwseftlqtkllavatLvvSllmsgl

TMH1-----------

6803sll0698 48 TFWAVNTIQEDAQLVDTRFGRDVGLLLAANVAPMIADKNLTEVARFSSRFYENT-SNIRY
cwat197946 41 TFWAVNTIQKDAQLNDTRFGRDLGLLLASNVTPLIAEDDLTEVARFSSRFYQST-TSIRY
7120alr3511 41 TFWAVNTIQQDAQVNDTRFGRDLGLLLAANVAPLVADHNLTEVAQFSQRFYSST-SSVRY
AvarZP00161981 41 TFWAVNTIQQDAQVNDTRFGRDLGLLLAANVAPLVADHNLTEVAQFSQRFYSST-SSVRY
NPUNNpR3716 41 TFWAVNTIQQDARMNDTRFGRDLGLLLAANVAPLVADNNLTEVAQFSQRFYSST-SSVRY
TERY00073906 41 SFWAVNTIQEDARLNDTRYGRDLGLLLAANVAPLMAEGNRNEVARFSRLFYKST-SSVRY
7942126282 41 TFWAVNSIQEDARLNDTRFGRDLGLLLAANATPLIADEDIAGLTRFSYRFYRST-SSVRY
TBP1tlr0437 49 TFWAVNTIQTNAHLNDTRYGRDLGLLLAADVAPLVAKGDTAAVAEFSRKFYERS-ASIRY
SS12Pro1422 60 TFFALSSIQRDAGMNDTRYARDLGLLLSGNVTELVAKGQERELFNVAEKFWRSS-RNIRY
MED4PMM1341 60 TFFALNSIQRDAGMNDTRYARDLGLLLSGNVTELVANNQRKEISNVAEKFWRSS-RNLRY
960533/318 60 TFFALNGIQRDAVMNDTRYARDLGLLLAGNVTELVAQGQDRELANVAEKFWRSS-RSVRY
990231/129 60 TFFALAGIQRDAAMNDTRYARDLGLLLAGNVTELVADGQDRELANVAEKFWRSS-RSVRY
SYNW0551 57 TFLALNGIQRDAVMNDTRYARDLGLLLAGNVTELVAQEQDRELANVAEKFWRSS-RSVRY
9313PMT1417 60 TFFVLNGIQRDAGMNDTRYARDLGLLLSGNVTELVAQGRDRELATVAEQFWRS--RSLRY
7421gll0055 54 TFWALNYIQEDARRSDQRFGEAVGNLLAATAAPLIALDQYPQLGEFTEEFLASNADNILY
Ppurpurea 56 TFWTLTSIQQETRLIDNRFGKDLSLLLAVNITPILEGDNYLQLQQFIEHFYLST-SSIRY
Gtenuistipitata 37 TFWSLTMLQEDLMIADKRFCKDLGFLLASSIIDSSEMNKQKDIAYFLEKIYLST-ASIRY
consensus 61 tFwalntiQ da lnDtRfgrdlglLLaanvtplva n evaqfserfy st sirY

----TMH1 PSD-----------------------------------------------

6803sll0698 107 MIYADPSGKIFFGIPYSEE--TVQNSL-TLERRIELPQIDPH--NFDQ----PFVRQHHT
cwat197946 100 IIYTDKTGNIFFGIPYSSA--EVQNSL-TIERRIQLPDFPSD--DHNL----PLVRQHQS
7120alr3511 100 MLYADETGKIFFGIPFWEP--EVENSL-SIERRIQLPEEYPG--DGEK----PMVRQHMS
AvarZP00161981 100 MLYADETGKIFFGIPFWEP--EVENSL-SIERRIQLPEEYPG--EGEK----PMVRQHMS
NPUNNpR3716 100 MLYADETGEIFFGIPFWEA--EVENSL-TIKRRIQLPEDYPG--DGEK----PMVRQHMT
TERY00073906 100 MLYADEEGKIFLGIPFSES--EVKTSL-TLRRRIQLPEDFMANIEDEKYSALPLVRQHLT
7942126282 100 LLYADTEGEIYLGIPFSQS--EVRNSL-TIQRRIQLPPDYLR--QGDR----PYVRQHLT
TBP1tlr0437 108 ILYADPDGEIYYGLPYSAP--QVESAL-TLRRRIQLPETYHA--SQE-----PLVRQHQT
SS12Pro1422 119 IFFTDTEGIVQLGIPISATPSDSKSEF-QLRKKLQLPQELKK--RPQF----PLVRQHLT
MED4PMM1341 119 IFFTDADDIVQLGIPISATPTSSDNQF-QLTRRLKLPSELKK--RPQF----PLVRQHAT
960533/318 119 IFFADPEGVVYLGIPISATPSSGDGEL-RLNRRLELPDELRR--RPQN----PLVRQHLT
990231/129 119 IFFADPEGVIYLGIPISATPNGEDGEL-RLNRRLELPDELRR--RPQN----PLVRQHLT
SYNW0551 116 IFFADPDGVVYLGIPISGAAAGGDGDL-RLNRRLELPDELRR--RPQN----PLVRQHLT
9313PMT1417 118 IFFADPDGLIYLGIPISASPVSGDNDL-QLNHRLELPAELPS--RPEN----ALIRQHLT
7421gll0055 114 VLYADSKGDILYGSPFNTNDTRANAPVPALIRHIELPPVLPA------------KRQHRT
Ppurpurea 115 ILVFNADGQIYYSIPFSSETAINFFSLSEYNCFRNENHYFSN---------TPIVNTNNR
Gtenuistipitata 96 ILYFDQNGSLLLGLPIYNTKIQNILQLHQNLLQLDNKEFLFN---------TPLINSSKL
consensus 121 ilyad eg iflgiPys e l l rriqlpde plvrqhmt

------------------------------------------------------------

6803sll0698 158 PNGDVTDVFIPLQYQGKFLGVLAIGINPNPAAVNSSNLTRDVTIAVFISIWVMVILGAVF
cwat197946 151 PNGEVTDVFVPLRHQGNKLGFLSIGINPNATVVNSSNLTRDVTIAVFVAIWAMVILGAVF
7120alr3511 151 PDGIVTDVFIPLIVDKKYLGVLAIGINPNQTAVISTNFTRDVTIAVFITIWVMVILAGVV
AvarZP00161981 151 PDGTVTDVFIPLIADKKYLGVLAIGINPNQTAVISTNFTRDVTIAVFITIWVMVILAGVV
NPUNNpR3716 151 PDGVVTDVFIPLIVNKKYLGVLAIGINPNQTAVISTNFTRDVTIAVFITIWVMVILAGVI
TERY00073906 157 PNGQLTDVFVPLIDESKYLGVLAVGINPNPTVVVSSNLTRDVTLAVFVSIWAMVILGAVV
7942126282 151 PAGLVTDVFVPLFQNDRYLGVLAIGTNPSPAVVASSGLTRDVTIAVFITIWVMVIVGAVF
TBP1tlr0437 158 PNGLVADVFVPLTFNGENLGVVALGINPNPTLIASANLTRDLTIAVFVSLWIMVILGGVF
SS12Pro1422 172 PQGQVTDVFVALLFKGKYVGNLALGVTPNKSALASAALTREITIAVFISIWILVIIGAVF
MED4PMM1341 172 PQGQVTDVFVPMLWKGKYLGTLALGVTPNKKALSSAALTREVTIAVFISIWVLVILGAVF
960533/318 172 PQGAVTDVFVPLIRGGQYYGVLGLGVNPNETALASAALTREVTVAVFISIWVLVILGAVF
990231/129 172 PQGTVTDVFVPLIRSGTYYGVLGLGVNPNETALASAALTREVTVAVFISIWVLVILGAVF
SYNW0551 169 PQGRVTDVFVPLIRNGRYYGVLGLGVNPNETALASAALTREVTVAVFISIWVLVILGAVF
9313PMT1417 171 PQGQLTDVFVPLVWKGDYLGTLALGVNPNDTALASAALTRQVTIAVFISIWVLVILGAVF
7421gll0055 162 PRGLVTDIFVPIEYGERQLGVVLVGINPEANLVSRSDLTRDVTVAVFVLVWIIAILGSVF
Ppurpurea 166 LQGEVIDIIIPLSKEKKLLGILNIGINSNPTLTTSSQLTRDVSVAVFISIWLMVILGAAF
Gtenuistipitata 147 LNNNIIDIIIPLTKAGHNLGSLDLGIDVN-IRFSSSRLIRDLSIFVFVLVWLMLFIGVAF
consensus 181 png vtDvfvpli gkylGvlaiGinpn tav ss ltRdvtiaVFisiWvmvilgavf

----------------------------------------PSD.TMH2/HAMP-------

6803sll0698 218 NALTITQPIKELLLGVKNIAAGNFKQRITLPFGGELGELIVNFNEMAERLERYEAQNIEE
cwat197946 211 NALTITRPIKELLLGVKNIAAGNFKQRINLPFGGELGELIFSFNEMAKRLERYEEQNIDE
7120alr3511 211 NALTITKPIKELLVGVKQIAAGNFKQRIDLPLGGELGELILNFNEMAERLERYEEQNIEE
AvarZP00161981 211 NALTITKPIKELLVGVKQIAAGNFKQRIDLPLGGELGELILNFNEMAERLERYEEQNIEE
NPUNNpR3716 211 NALTITKPIKELLVGVKEIATGNFKQRIDLPLGGELGELILSFNEMAERLERYEEQNIEE
TERY00073906 217 HALIITKPIKKLSEGVKNIAQGNFKQRIDLSFGRELGELILSFNEMAEQLENYKKQNIEK
7942126282 211 NALIITRPIRELVEGVRSIASGNFKQRIDLPPSGELGELIASFNDMAEQLEGYAAQNIDE
TBP1tlr0437 218 NALTITQPIKELVQGVKNIAAGNFKQRINLPFGGELGELITSFNDMAERLASYEAQNIEE
SS12Pro1422 232 NAQTITKPIRELVSGVREIAKGNFKSRIILPMSGELGELLDGFNNMASQLEDYDAANIEE
MED4PMM1341 232 NALTITRPVRELVKGVREISKGNFKSRISLPMTGDLGELLTGFNRMASQLENYDEANIEE
960533/318 232 NALTITRPVKELLRGVRSVASGNFGARVDLPVGGELGELLTGFNAMASQLEAYDEANIEE
990231/129 232 NALTITRPVKELLRGVRSVASGDFQARVDLPMGGELGELLTGFNAMASQLEAYDEANIEE
SYNW0551 229 NALTITRPVKELLRGVRSISLGNFQARIDLPVGGELGELLTGFNAMASQLEAYDEANIEE
9313PMT1417 231 NALTITQPVKELLRGVRAIASGNFEARIALPMGGELGELLNGFNAMASQLEAYDAANIEE
7421gll0055 222 NALTITQPLKELVQGVQRIAQGNFKQRVTLAYPGEIGELITSFNLMAQRLQSYEEQNIEE
Ppurpurea 226 NAFTITRPIRELLTGVKNIASGDFYQRIDLPFGGELGALIFNFNEMAERLEKYEQQNVEK
Gtenuistipitata 206 NTLSVATPQKKLLLGIQNIASGNFNQRLTVPVNYQLSTLIISFNEMAEKLQSYEKKNVDK
consensus 241 naltitrPikeLl Gvk ia GnFkqRidlplggelgeLi FNeMAerLe YeeqNiee

-----------TMH2HAMP----------------------------HAMP

6803sll0698 278 LTAEKAKLDTLVSTIADGAMLVDTNLQLLLVNPTARRLFAWENKP-IIGENLLENLPPEI
cwat197946 271 LTSEKAKLDTLVSTIVDGAILLDTNLNLLLVNPTARRMFVWDSKN-VIGENILEILPSEL
7120alr3511 271 LTAEKAKLETLVSTIADGAVLIDNNMQVILVNPTARHIFGWEGADDVVGSNVLHHLPPAV
AvarZP00161981 271 LTAEKAKLETLVSTIADGAVLIDNNMQVILVNPTARHIFGWEGADDVVGSNVLHHLPPTV
NPUNNpR3716 271 LTAEKAKLETLVSTIADGAVLIDNNMQVILVNPTARRIFGWETAD-VVGCNVLHHLPPAV
TERY00073906 277 LTAEKAKLETLVSTIADGALLLDANLQLILVNPTARRIFSWEEKK-VIGGKVTDNLPVAV
7942126282 271 LTSEKAKLETLVSTIADGALLLDADLNLVLVNPAAIRIFGWEGRS-LKGDDVIDHLPSPL
TBP1tlr0437 278 LQAEKAKLDTLVSTIADGAILLDTDMRIILVNPTAQRLFNWEGMN-VIGQNALDCFPAPV
SS12Pro1422 292 LRAAQVKQQSLIATMADGAILLNEKGKIVLVNPTARRLFRWEGRN-LESQDLLDELPELI
MED4PMM1341 292 LKAAQIKQQSLIATMADGAILLDSKGKIVLTNPTAKRLFRWEGRF-LEGKYLLNEIPEIL
960533/318 292 LTAAQVKQQSLIATMADGAMLLDADGRIVLANPTARRLFRWEGRS-LEGQELVGELPELL
990231/129 292 LTAAQVKQQSLIATMADGAILLDATGRIVLANPTSRRLFRWEGRS-LEGQELVEELPELL
SYNW0551 289 LTAAQVKQQSLIATMADGAILLDEQGRIVLANPTARRLFRWEGRK-LEGQELVAELPDLL
9313PMT1417 291 LTAAQVKQQSLIATMADGALLLDEVGQIVLANPTARRIFRWEGRN-LEGQELLNELPEII
7421gll0055 282 ITAEKAKLETFIATIADGGILLDSSLRILLVNPAMLRIFGWGSRV--VGKNILDLLPVGM
Ppurpurea 286 LTSEKAKLETLVSTIADGAILLDKDLRVILVNRTAIENFGWEGKN-IAGSIIVDYLPEDI
Gtenuistipitata 266 IISEKTKLETIVSLIAEGTILIDAELRILFVNKKAQEIFNWFNID-LTGGYICSYLPIHI
consensus 301 ltaekaKldtlvstiadGaiLld lrillvNptarrlF Wegk i g lle lP i
 PAS----------------------------------------------------

6803sll0698 337 TAQLTQPLRELAADQGSL---LFSPGHGPQEEEQDKTYAPEEFRISLTQPFPRTIRLMLT
cwat197946 330 TEQLQTPLKQMMEQNTSVSDELESEYQTAEDTPLQPRISPGEYRISISQPISRIVRVLLT
7120alr3511 331 QMEITRPLYEMAAG----------------------ECDSAEFRLTLNQPSNRTIRILLT
AvarZP00161981 331 QMEITRPLYEMAAG----------------------ECDSAEFRLTLNQPSNRTIRILLT
NPUNNpR3716 330 QMEITGPLYEMAAG----------------------ECESAEFRIFINQPTPRTIRILLT
TERY00073906 336 QMELNRPLYQIATG----------------------EIEGGEYRCTLDEPNKRTIRVLLT
7942126282 330 AQELHDPLKAIANG----------------------DREAVELRLTLPERSNRTVRILVS
TBP1tlr0437 337 CEKLTCPLYKASRG----------------------ESEGGEFRVTLQEPSSRSVRILLT
SS12Pro1422 351 ANELHPNITSILNN----------------------LCESDELRCSTGEP-ARTLRIVLQ
MED4PMM1341 351 SNDLHTNIESILKR----------------------EKENDDLRCSLGEP-ARTLRIVLQ
960533/318 351 AIELHSPLDALLGG----------------------GSDSEDLRCSVGEP-ARTLRIVLQ
990231/129 351 SIEVHSALDALLFS----------------------SSDSEDLRCSVGEP-ARTLRIVMQ
SYNW0551 348 AIELQAPLDLLLIS----------------------GADSEDLRCSVGEP-SRTLRIVMQ
9313PMT1417 350 ANELHDPLQSLLRN----------------------IGESNDLRCSLEEP-SRTLRIVLQ
7421gll0055 340 RAEVEEPLDSVSRN----------------------ILEQAECRVVLDKR---TLRVLIS
Ppurpurea 345 NQQLFPILNDIIRKN----------------FLEQSICETQEICIKLQKNYKKTFRVLLT
Gtenuistipitata 325 NEALLPILNNLVQSSYISTN----------------KSQTEEICINLDYNSRKICRFLLT
consensus 361 el pl lm es elrisl ep rtiRillt
 -----PAS

6803sll0698 394 QVLDQN---------------------------------------------RENLRGIVM
cwat197946 390 QVFDQH---------------------------------------------RETLKGIAM
7120alr3511 369 TVLNLQ---------------------------------------------RESIKGIAI
AvarZP00161981 369 TVLNLQ---------------------------------------------RESIKGIAI
NPUNNpR3716 368 TVLNVQ---------------------------------------------RESIKGIAI
TERY00073906 374 TVLLYRASGTESLCKSCIYDAYDSCSSANRPVVYECTLYENKLGTDFISPYRNSLKGIAM
7942126282 368 NVLDTA---------------------------------------------RANVKGIAV
TBP1tlr0437 375 TVMDVQ---------------------------------------------REKPKGIAI
SS12Pro1422 388 SVRDSS---------------------------------------------GSTLKGVAI
MED4PMM1341 388 SVLDTN---------------------------------------------KVELKGIAV
960533/318 388 AVRDAS---------------------------------------------GETLKGIAV
990231/129 388 AVRDAS---------------------------------------------GESLKGIAV
SYNW0551 385 AVRDAS---------------------------------------------GESLKGIAV
9313PMT1417 387 SVRDQS---------------------------------------------GETLKGIAI
7421gll0055 375 PVLAPRG--------------------------------------------DNLLKGVVL
Ppurpurea 389 TVLDHK---------------------------------------------YSILKGIAM
Gtenuistipitata 369 TLLDRI---------------------------------------------LKVLTGVVI
consensus 421 tvld retlkGiam


6803sll0698 409 TVQDITREVELNEAKSQFISNVSHELRTPLFNIKSFIETLSEFGEDLSEVERKEFLETAN
cwat197946 405 TVQDITREVELNEAKSQFISNVSHELRTPLFNIKSFIETLSEFGEDLTETERKEFLETAN
7120alr3511 384 TVQDITREVELNEAKSQFISNVSHELRTPLFNIKSFIETLHDYGEDLSLEERQEFLQTVN
AvarZP00161981 384 TVQDITREVELNEAKSQFISNVSHELRTPLFNIKSFIETLHDYGEDLSIEERQEFLQTVN
NPUNNpR3716 383 TVQDITREVELNDAKSQFISNVSHELRTPLFNIKTYIETLHDYGEDLGLEERQEFLQTVN
TERY00073906 434 TVQDITREVELNDAKSQFISNVSHELRTPLFNIKSFIETLHEYGEDLSEVERREFLETAN
7942126282 383 TVQDITREVELNEAKSQFISNVSHELRTPLFNIKSFIETLHDYGDDLSEEERREFLSTAN
TBP1tlr0437 390 TIQDITREVELNEAKAQLISNVSHELRTPLFNIKSIIETIQEYGSSLSEKEQQEFLETAN
SS12Pro1422 403 TIQDLTREVELNAIQRRFISNVSHELRTPLFNIKSYVETLHDLDDKLDHKEKMEFLEVAN
MED4PMM1341 403 TIQDLTREVELNAAQNRFISNVSHELRTPLFNIKSYVETLYDLKDQLSNEEQLEFLGIAN
960533/318 403 TIQDLTREVELNAAQSRFISNVSHELRTPLFNIKSYVETLHDLGDQLSPDEQKEFLGVAN
990231/129 403 TIQDLTREVELNAAQSRFISNVSHELRTPLFNIKSYVETLHDLGDQLTEDEKKEFLGVAN
SYNW0551 400 TVQDLTREVELNAAQSRFISNVSHELRTPLFNIKSYVETLHDLGDQLSPEEHKEFLGVAN
9313PMT1417 402 TVQDLTREVELNAAQSRFISNVSHELRTPLFNIKSYVETLHDLGDQLSEEEKKEFLGIAN
7421gll0055 391 TVQDLSKEAELNQAKSQFISNVSHELRTPLSSIKSYIETVYEFGDSLDDSTKNEFLKTAN
Ppurpurea 404 TIQDRTQEVELNEIKNQFISNVSHELRTPLFNIRSFLETLYEYHDSLDDSQKLEFLAIAN
Gtenuistipitata 384 IIQDISKEAKLNEAKNQFIGNISHELRTPLCNIGSFLETLIDYNSTLKEKEKINFLTIAN
consensus 481 tvQDitrEveLNeaksqfIsNvSHELRTPLfnIksfiETlhdfgd Lseeer eFL taN
 HisKA-------------------------------------------

6803sll0698 469 HETDRLSRLVNDVLDLSKLESSKIYQLDAVDLYQLIEQSLRSYQLNAKDKQLQLEKILDP
cwat197946 465 HETDRLTRLVNDVLDLSRLESSKTYHLKGIDLSQLIEQTLRSYQLNAKDKELVLKKEVEP
7120alr3511 444 HETDRLTRLVNDVLDLSKLESGRSYSFDGVDLAQAIEQTLRTYQLNARDKGIELIQDVAP
AvarZP00161981 444 HETDRLTRLVNDVLDLSKLESGRSYSFDGVDLAQAIEQTLRTYQLNAKDKGIELIQDVAP
NPUNNpR3716 443 HETDRLTRLVNDVLDLSKLESGRQYNFDGVDLAQAIEQTLRTYQLNAKDKGVELLQEVDT
TERY00073906 494 HETDRLTRLVNDVLDLSRLESCMIYHLNAVDVAQPIEQTLRTYHLNAKDKKIELNYDIES
7942126282 443 HETDRLTRLVNDVLDLSRLESNRRYNFEGIDLVQPIEQTLRTYQLNAREKGIELRSEIAR
TBP1tlr0437 450 HETDRLTRLVNDFLDISRLESGRPYQFGSVQMAQVIDQIMRTYQLNAANKSITLTAEVEN
SS12Pro1422 463 SETDRLTRLVNDVLDLSKIETAGNVNFEAINLAPAIDQTVRTYRLNAEDKKVEISQEIEE
MED4PMM1341 463 SETDRLTRLVNDVLDLSRLESGKIIQLEPMEIKPAIEQTLRNYRLNATEKNVSLAHDIEE
960533/318 463 DETDRLTRLVNDVLDLSRLESGRTLQFEPISMRPAMEQTLRTYRLNAEDREVELVLDVPE
990231/129 463 DETDRLTRLVNDVLDLSRLESGRTVQFETMNLRPAMEQTLRTYRLNAEDKTVELELDVAP
SYNW0551 460 DETDRLTRLVNDVLDLSRLESGRAVQFEAMNLLPAMEQTLRAYRLNADDKHVKLELDAPE
9313PMT1417 462 SETDRLARLVNDVLDLSRLESGRTVQFEPMDLRPAIEQTLRNYRLNADDKQIHIELNTDD
7421gll0055 451 QETDRLTRLVNDVLDLSRLESGREYHFEPVDLVQPIEQTLRTHRLTARDREIELVSDIAT
Ppurpurea 464 KETGRLTRLVNDVLDLSRLESDQEYTLQPTDLVSAVEQTIRTYQLSAKDKRIDLHIDIEQ
Gtenuistipitata 444 NETKRLSSLVNDILDLSVLESEHDYKLDYIDLTQILYNVVNTFQITANKNNIRLIVELEK
consensus 541 hETdRLtrLVNDvLDlSrlEsgr yqfdavdl qaieqtlrtyqlnAkdk iel dld
 ------------------HisKA

6803sll0698 529 DLPFALGNYDLLLQVMTNLIGNSFKFTKAGGKIIVRAYPLHR------------------
cwat197946 525 NLPLILGHYDLLLQVMTNLVGNSLKFTPSKGMIVIRAYHFKT------------------
7120alr3511 504 GLPLVLGNYDLLLQVLANLVGNALKFTSSGGKIAIRTYVLN-------------------
AvarZP00161981 504 GLPLVLGNYDLLLQVLANLVGNALKFTPSGGKIAIRTYVLH-------------------
NPUNNpR3716 503 NLPLVMGNYDLLVQVFGNLIGNALKFTKAGGKVAIRAYQLD-------------------
TERY00073906 554 NLPTVVGHYDLLLQVFANLVGNAMKFTEPGGRIVIRAYLLESDIEIEQIEQGGWIVSPPP
7942126282 503 DLPAVRANYDLLLQVFANLVGNALKFTASGGCVAMRAYLVGQPLR--------------T
TBP1tlr0437 510 PLPPVWGNYDLLIGALTNLVGNALKFTPENGRVTIRAYVWHP------------------
SS12Pro1422 523 NMELILGNWDLLLQVLDNLIGNALKFSSAGGKIVLRAYTWPDTCITSSPKIDKK------
MED4PMM1341 523 NIPSILGNFDLLLQVFDNLLGNGLKFSPKNSTLKIRAYTWPDSCPAFPPNNNK-------
960533/318 523 DLPDVLGNWDLLLQVLDNLMGNALKFSRPGGPLALRAYPWPDTCSVEGTAITGT------
990231/129 523 DLPDVLGNWDLLLQVFDNLVGNALKFSRPGGILSMRAYVWPDTCRVEAPISTKS------
SYNW0551 520 DLPEVLGNWDLLLQVLDNLVGNALKFSRSGGTLALRAYPWPDTCPVGSPNDEQ-------
9313PMT1417 522 ELPTILGNWDLLLQVLDNLVGNGLKFSRAGGSLMVRAYTWPDSCKMS-PIESSK------
7421gll0055 511 DLPLVLGNYDLLQQVFSNLVGNALKFTEPGGRVTLSARRTDD------------------
Ppurpurea 524 NLQCVLGNYNLILQILANLVVNSLKFTHPNGIIILRAYTVDDLKT---------------
Gtenuistipitata 504 NITYVFAHESSILQVISNLLNNALKFSPYHSLIIVRVYKLICNN----------------
consensus 601 lp vlgnydlllqvm NLvgNalKFt gg i vray
 HATPase_c---------------------------------------------

6803sll0698 571 ---SNLRAEDGPGLVRVEISDTGIGIDPEDQAAIFERFYRVENRVHTLEGTGLGLSIVKN
cwat197946 567 ---KSKQFNN-KSCVRVEISDSGIGIAPEDQAAIFDRFFRVENRVHTLEGTGLGLSIVRN
7120alr3511 545 ---TKSNSPNQPAQVRIEVSDTGIGIAQEDQQAIFDRFFRVENRVHTLEGTGLGLSIVRN
AvarZP00161981 545 ---TKPNSPNQPTQVRIEVSDTGIGIAPEDQQAIFDRFFRVENRVHTLEGTGLGLSIVRN
NPUNNpR3716 544 ---FKPN-HSQSSPVRIEISDTGIGIATEDQHSIFERFFRVENRVHTLEGTGLGLSIVRN
TERY00073906 614 PRPTELNLKSNNQFVRIEISDTGSGIEPEDQEAIFERFFRVENRVHTLEGTGLGLSIVRN
7942126282 549 EAVNHSEIVSSLPNIRIEINDTGIGIDAEDQEAIFDRFFRVENRVHTLEGTGLGLSIVRN
TBP1tlr0437 552 ------SSDPEQERVRIEVADTGMGIAPEDQPRVFERFFRVENRVHTLEGTGLGLAIVQD
SS12Pro1422 577 -DAPSCEIIYPLPRLRIEIGDTGCGISEDSQLRIFERFYRVENDVHTEVGTGLGLSIVRE
MED4PMM1341 576 -DAPQCELVSPLPKVRIEIADNGSGISQPDQEKIFDRFYRVENAVHTEQGTGLGLSIVRG
960533/318 577 -DGPTCALTSPLPKLRVEIADTGCGISSTDQERIFDRFFRVENAVHTEVGTGLGLSIVRG
990231/129 577 -EGPTCTLTSPLPRLRVEIADTGYGISAQDQQRIFDRFYRVENAVHTEVGTGLGLSIVRG
SYNW0551 573 -AGPSCALSSPLPRLRVEVADTGCGISAADQERIFDRFFRVENAVHTEVGTGLGLSIVRG
9313PMT1417 575 -SAPHCEFFSPLPKLRVEVADTGHGINQDDQQHIFDRFYRVENAVHTEAGTGLGLSIVRG
7421gll0055 553 -----------PAKVRVAVTDTGIGISEEDQRRIFERFFRVENRVHTLEGTGLGLSIVDN
Ppurpurea 569 ---ETEVQHFNSQKVRVEICDNGIGISRKNQERIFARFLRIENYVHTLEGTGLGLSIVKN
Gtenuistipitata 548 ---TFTSHIHNRDLVRVEILDQGIGIAEEDQKIIFDRFVRVENNIHTLEGTGLGLSIVKN
consensus 661 rvRvei DtGiGI edQ riFdRFfRvENrvHTleGTGLGLsIVrn
 ------------------------------------------------------------

6803sll0698 628 IIAKHQSQIHLVSEVGVGTTFWFDLAVYQS-------------MLMVVG-----------
cwat197946 623 IIDKHHSRINLISEVGIGTTFWFDLALYEDNCTPSEGCTNDNILPRTVDVHPNSLLF---
7120alr3511 602 IVERHRSRVNLVSEVGVGTTFWFDLILFEDEAPKKMVESGEEKAAMPTS-----------
AvarZP00161981 602 IVERHRSKVNLVSEVGVGTTFWFDLILFEDEAPKKMAESGEEKAALPAN-----------
NPUNNpR3716 600 IIDRHRSKVHLVSEVGIGTTFWFDLAAFEEKAPPIQVEAITEASKITTV-----------
TERY00073906 674 IIDKHHSKVNLVSELGVGTTFWFDLAVFEERSVPEENQLLEVP-----------------
7942126282 609 IVEKHQSRIHLISEVGIGTTFWFDLPIYQPEEAIAAPAKPEAITATNGTEVSVSTG----
TBP1tlr0437 606 IIHKHNTQIHLISELGVGSTFWFDLAIDESALVDNPDGRAETALPPA-------------
SS12Pro1422 636 IIEKLGSNIRMVSIPGIGTTFWFDLPLAINDADEILVESERMRLKWDRKLEEELT-----
MED4PMM1341 635 IIEKHGGQIRMASELGIGTTFWFDLPLEQSDKDELLAQTINNVENFSDSQVSELF-----
960533/318 636 ILEKHGAQVQMASEPEVGTTFWFDLPLAKADKDELQLQAERRSRNAIAEAVEL-------
990231/129 636 ILDKHGAQVSMVSELDVGTTFWFELPLEQADRDELALQAERRSISAASDDGNIESVAVGS
SYNW0551 632 ILEKHGTKVSMASEPEVGTTFWFDLPLGQADVDELKLQAERRSTAEQLA-----------
9313PMT1417 634 IIEKHGGQIRMASEVELGTTFWFDLPLEQTDADELIVQSVRTTRQQEQGLEL--------
7421gll0055 602 IVKKHNAQVHIESEVGQGSTFWFDVEAYMETCEWPRREQEEVI-----------------
Ppurpurea 626 IIQKHNSEIHLYSELKNGSCFFFDLMIAKDE-----------------------------
Gtenuistipitata 605 ILAKYKISVNVQSQLNVGTSIWFNLKYVS-------------------------------
consensus 721 Iidkh s vhlvSevgvGttfwFdl l e
 ------------------HATPase_c

**ClustalW alignment of NblS (DspA, Ycf26) putative amino acid sequences.**

6803 - *Synechocystis sp.* PCC 6803, cwat - *Crocosphaera watsonii* WH 8501, 7120 - *Anabaena sp.* PCC 7120, Avar - *Anabaena variabilis* ATCC 29413, NPUN - *Nostoc punctiforme* PCC 73102, TERY - *Trichodesmium erythraeum* IMS101, TBP1 - *Thermosynechococcus elongatus* BP-1, 7942 - *Synechococcus elongatus* PCC 7942, 7421 - *Gloeobacter violaceus* PCC 7421, Ppurpurea – *Porphyra purpurea*, 9313 - *Prochlorococcus marinus* MIT9313, SYNW - *Synechococcus sp.* WH8102, MED4 - *Prochlorococcus marinus* MED4, 9605 – *Synechoccocus CC9605*, 9902 – *Synechococcus CC9902*, SS12 - *Prochlorococcus marinus* SS120, Gtenuistipitata - *Gracilaria tenuistipitata*, Ppurpurea – *Porphyra purpurea*. Also indicated are the positions of the domains (see Figure 1).
